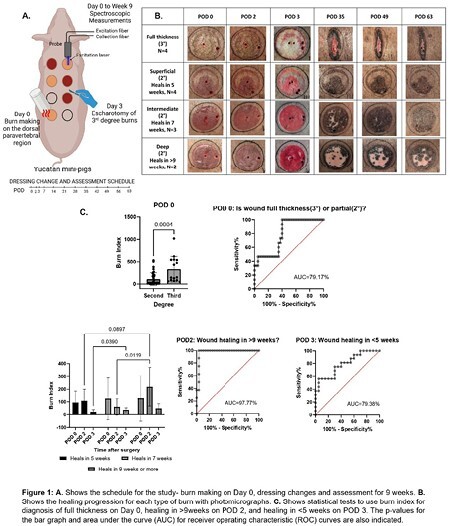# 133 Early Assessment of Burn Depth in a Yucatan Minipig Model Using Resonance Raman Spectroscopy

**DOI:** 10.1093/jbcr/irae036.132

**Published:** 2024-04-17

**Authors:** Rohil Jain, Yanis Berkane, Emmanuella O Ajenu, Khanh T Nguyen, Austin A Shamlou, Alexandre G Lellouch, Basak E Uygun, Curtis L Cetrulo, Jr., Mark A Randolph, Korkut Uygun, Padraic Romfh, Shannon N Tessier

**Affiliations:** Shriner's Childrens' Hospital, Massachusetts General Hospital, Harvard Medical School, Boston, Boston, Massachusetts; Shriner's Childrens' Hospital, Massachusetts General Hospital, Harvard Medical School, Boston, Cambridge, Massachusetts; Massachusetts General Hospital, Boston, Massachusetts; Harvard Medical School, Boston, Massachusetts; Massachusetts General Hospital and Shriners children’s Boston, Boston, Massachusetts; Massachusetts General Hospital, Harvard Medical School, Boston, Massachusetts; Shriners- Boston, Boston, Massachusetts; Pendar Technologies, Palo Alto, California; Shriner's Childrens' Hospital, Massachusetts General Hospital, Harvard Medical School, Boston, Boston, Massachusetts; Shriner's Childrens' Hospital, Massachusetts General Hospital, Harvard Medical School, Boston, Cambridge, Massachusetts; Massachusetts General Hospital, Boston, Massachusetts; Harvard Medical School, Boston, Massachusetts; Massachusetts General Hospital and Shriners children’s Boston, Boston, Massachusetts; Massachusetts General Hospital, Harvard Medical School, Boston, Massachusetts; Shriners- Boston, Boston, Massachusetts; Pendar Technologies, Palo Alto, California; Shriner's Childrens' Hospital, Massachusetts General Hospital, Harvard Medical School, Boston, Boston, Massachusetts; Shriner's Childrens' Hospital, Massachusetts General Hospital, Harvard Medical School, Boston, Cambridge, Massachusetts; Massachusetts General Hospital, Boston, Massachusetts; Harvard Medical School, Boston, Massachusetts; Massachusetts General Hospital and Shriners children’s Boston, Boston, Massachusetts; Massachusetts General Hospital, Harvard Medical School, Boston, Massachusetts; Shriners- Boston, Boston, Massachusetts; Pendar Technologies, Palo Alto, California; Shriner's Childrens' Hospital, Massachusetts General Hospital, Harvard Medical School, Boston, Boston, Massachusetts; Shriner's Childrens' Hospital, Massachusetts General Hospital, Harvard Medical School, Boston, Cambridge, Massachusetts; Massachusetts General Hospital, Boston, Massachusetts; Harvard Medical School, Boston, Massachusetts; Massachusetts General Hospital and Shriners children’s Boston, Boston, Massachusetts; Massachusetts General Hospital, Harvard Medical School, Boston, Massachusetts; Shriners- Boston, Boston, Massachusetts; Pendar Technologies, Palo Alto, California; Shriner's Childrens' Hospital, Massachusetts General Hospital, Harvard Medical School, Boston, Boston, Massachusetts; Shriner's Childrens' Hospital, Massachusetts General Hospital, Harvard Medical School, Boston, Cambridge, Massachusetts; Massachusetts General Hospital, Boston, Massachusetts; Harvard Medical School, Boston, Massachusetts; Massachusetts General Hospital and Shriners children’s Boston, Boston, Massachusetts; Massachusetts General Hospital, Harvard Medical School, Boston, Massachusetts; Shriners- Boston, Boston, Massachusetts; Pendar Technologies, Palo Alto, California; Shriner's Childrens' Hospital, Massachusetts General Hospital, Harvard Medical School, Boston, Boston, Massachusetts; Shriner's Childrens' Hospital, Massachusetts General Hospital, Harvard Medical School, Boston, Cambridge, Massachusetts; Massachusetts General Hospital, Boston, Massachusetts; Harvard Medical School, Boston, Massachusetts; Massachusetts General Hospital and Shriners children’s Boston, Boston, Massachusetts; Massachusetts General Hospital, Harvard Medical School, Boston, Massachusetts; Shriners- Boston, Boston, Massachusetts; Pendar Technologies, Palo Alto, California; Shriner's Childrens' Hospital, Massachusetts General Hospital, Harvard Medical School, Boston, Boston, Massachusetts; Shriner's Childrens' Hospital, Massachusetts General Hospital, Harvard Medical School, Boston, Cambridge, Massachusetts; Massachusetts General Hospital, Boston, Massachusetts; Harvard Medical School, Boston, Massachusetts; Massachusetts General Hospital and Shriners children’s Boston, Boston, Massachusetts; Massachusetts General Hospital, Harvard Medical School, Boston, Massachusetts; Shriners- Boston, Boston, Massachusetts; Pendar Technologies, Palo Alto, California; Shriner's Childrens' Hospital, Massachusetts General Hospital, Harvard Medical School, Boston, Boston, Massachusetts; Shriner's Childrens' Hospital, Massachusetts General Hospital, Harvard Medical School, Boston, Cambridge, Massachusetts; Massachusetts General Hospital, Boston, Massachusetts; Harvard Medical School, Boston, Massachusetts; Massachusetts General Hospital and Shriners children’s Boston, Boston, Massachusetts; Massachusetts General Hospital, Harvard Medical School, Boston, Massachusetts; Shriners- Boston, Boston, Massachusetts; Pendar Technologies, Palo Alto, California; Shriner's Childrens' Hospital, Massachusetts General Hospital, Harvard Medical School, Boston, Boston, Massachusetts; Shriner's Childrens' Hospital, Massachusetts General Hospital, Harvard Medical School, Boston, Cambridge, Massachusetts; Massachusetts General Hospital, Boston, Massachusetts; Harvard Medical School, Boston, Massachusetts; Massachusetts General Hospital and Shriners children’s Boston, Boston, Massachusetts; Massachusetts General Hospital, Harvard Medical School, Boston, Massachusetts; Shriners- Boston, Boston, Massachusetts; Pendar Technologies, Palo Alto, California; Shriner's Childrens' Hospital, Massachusetts General Hospital, Harvard Medical School, Boston, Boston, Massachusetts; Shriner's Childrens' Hospital, Massachusetts General Hospital, Harvard Medical School, Boston, Cambridge, Massachusetts; Massachusetts General Hospital, Boston, Massachusetts; Harvard Medical School, Boston, Massachusetts; Massachusetts General Hospital and Shriners children’s Boston, Boston, Massachusetts; Massachusetts General Hospital, Harvard Medical School, Boston, Massachusetts; Shriners- Boston, Boston, Massachusetts; Pendar Technologies, Palo Alto, California; Shriner's Childrens' Hospital, Massachusetts General Hospital, Harvard Medical School, Boston, Boston, Massachusetts; Shriner's Childrens' Hospital, Massachusetts General Hospital, Harvard Medical School, Boston, Cambridge, Massachusetts; Massachusetts General Hospital, Boston, Massachusetts; Harvard Medical School, Boston, Massachusetts; Massachusetts General Hospital and Shriners children’s Boston, Boston, Massachusetts; Massachusetts General Hospital, Harvard Medical School, Boston, Massachusetts; Shriners- Boston, Boston, Massachusetts; Pendar Technologies, Palo Alto, California; Shriner's Childrens' Hospital, Massachusetts General Hospital, Harvard Medical School, Boston, Boston, Massachusetts; Shriner's Childrens' Hospital, Massachusetts General Hospital, Harvard Medical School, Boston, Cambridge, Massachusetts; Massachusetts General Hospital, Boston, Massachusetts; Harvard Medical School, Boston, Massachusetts; Massachusetts General Hospital and Shriners children’s Boston, Boston, Massachusetts; Massachusetts General Hospital, Harvard Medical School, Boston, Massachusetts; Shriners- Boston, Boston, Massachusetts; Pendar Technologies, Palo Alto, California

## Abstract

**Introduction:**

Assessment of thermal burns in the acute phase is critical since healing may require surgical intervention depending on the depth of injury. Early depth estimation guides debridement and planning care strategy. Current assessment standard has limitations, and biopsies are invasive and painful. There is an urgent need for a rapid, non-invasive, and objective metric of burn depth.

**Methods:**

We created a model to study intermediate burn depth in Yucatan miniature pigs, which is clinically relevant. Brass blocks of 4cm radius create contact burns by preheating to 63°C for 2nd-degree (intermediate), and 95°C for 3rd-degree (full thickness) burns. Contact duration varied between 15 to 45 seconds for 2-degree burns to create different depths. Burns are evenly distributed and randomized on the dorsal paravertebral region of the pigs. Full-thickness burns were debrided on POD 3. Each wound was assessed using a custom bench-top Resonance Raman Spectroscopy (RRS) device to measure oxygen saturation, hemoglobin content, and autofluorescence spectrum. Measurements with RRS were performed immediately ( < 2 hours), 2 days, and 3 days after injury. Animals received punch biopsies and dressing changes every week for 9 wks and photographic documentation of healing. Post hoc analysis was performed in GraphPad Prism software to observe correlation between RRS readings on POD 0 to 3 and wound healing duration.

**Results:**

We successfully created intermediate and full-thickness burns, as confirmed by visual observation and histological analysis. We also created different depths of intermediate burns that vary in healing potential (>95% re-epithelialization). Of 9 wounds on 2 pigs, 4 healed in 5 wks, 3 in 7 wks, and 2 did not heal by 9 wks. RRS measurements had 4 technical replicates for each wound on POD 0, 2, and 3, and were correlated with wound type (2nd vs. 3rd) and duration of healing for 2nd-degree burns. We defined Burn Index (BI) as a marker of burn severity-

Burn Index B.I. = Oxygen Saturation / (Normalized Laser Induced Fluorescence * Normalized Hemoglobin Content)

BI distinguished between 2nd-degree (n=9) and 3rd-degree (n=4) burns on POD 0 with an area under the curve (AUC) for a receiver operating characteristic (ROC) of 79.17%. It could also distinguish wounds taking more than 9 wks to heal (n=2) from those that healed earlier (n=7) by POD 2, with AUC 97.77%, indicating high sensitivity and specificity. By POD 3, BI distinguished healing in less than 5 wks (n=4) from others (n=5) with AUC 79.38%.

**Conclusions:**

We established a model of intermediate burns with Yucatan miniature pigs. Resonance Raman Spectroscopy (RRS) based Burn index (BI) could distinguish full and intermediate thickness burns by POD 0 and predict healing time by POD 2 and POD 3 with high specificity and sensitivity.

**Applicability of Research to Practice:**

Rapid, early, non-invasive quantification of burn depth using our portable device can aid decision-making regarding intermediate-depth wound care, such as skin grafting.